# Protective role of grape seed proanthocyanidin antioxidant properties on heart of streptozotocin-induced diabetic rats

**Published:** 2015-06-15

**Authors:** Esrafil Mansouri, Layasadat Khorsandi, Amin Abdollahzade Fard

**Affiliations:** 1 *Cellular and Molecular Research Center, Department of Anatomical Sciences, Faculty of Medical Sciences, Ahvaz Jundishapur University of Medical Sciences, Ahvaz, Iran;*; 2 *Department of Physiology, Faculty of Medical Sciences, Urmia University of Medical Sciences, Urmia, Iran.*

**Keywords:** Diabetic heart, Grape seed proanthocyanidin, Oxidative stress

## Abstract

Grape seed proanthocyanidin (GSP) bears a very powerful antioxidant effects. Studies demonstrated that proanthocyanidins protect against free radicals mediated cardiovascular and renal disorders. The present study was designed to assess the effect of GSP on the heart of diabetic rats. Forty rats were divided into four groups of 10 animals each: Group I: control, Group II: control group were given GSP, Group III: diabetic group, Group IV: diabetic group treated with GSP. Diabetes was induced by a single dose of streptozotocin, and then GSP (200 mg kg^-1^ body weight) was administrated for four weeks. Blood glucose, glycosylated hemoglobin (HbA1c) and also the levels of lipid peroxidation and antioxidant enzymes were examined in the heart tissues of all groups. Oral administration of GSP to diabetic rats significantly reduced *(p < 0.05)* heart weight, blood glucose, HbA1c and lipid peroxidation level, but increased *(p < 0.05)* body weight and activities antioxidant enzymes when compared to diabetic group. The results indicated that GSP could be useful for prevention or early treatment of cardiac disorder caused by diabetes.

## Introduction

Diabetes is a chronic metabolic disorder characterized by a default of insulin secretion and/or increased resistance to insulin, and it is also associated with disturbance in metabolism of protein, lipids, carbohydrate and glucose.^1^ Investigates has indicated that enhanced oxidative stress is prevalent in chronic hyperglycemia.^2^ Hyperglycemia can produce oxidative stress via advanced glycation end products (AGEs) formation, elevated flux by the polyol pathway, enhanced activation of protein kinase C (PKC) and increased flux through hexosamine pathway.^3^ Oxidative stress is one of the main mechanisms of diabetic complications, and the imbalance in the formation of free radicles and defense system leads to more cellular injury that is common in diabetic people.^4^ Studies have expressed that oxidative stress is main factor of cardiovascular complications in diabetes mellitus.^5^ There is an inter-dependent process to defend the cells against oxidative injuries, such as the superoxide dismutase (SOD), glutathione peroxidase (GPx) and catalase (CAT) enzymes.^6^^,^^7^ In addition to the antioxidant enzymes, food antioxidants play a key role in supporting against the advancement of diseases caused by oxidative injuries.^8^ Nowadays interest has been concentrated on search of the defensive antioxidant biochemical activation of different plants and some natural products which demonstrated radical scavenger properties as well as ameliorated the antioxidant enzymes mechanism. In this regard, the protective features of flavonoids and polyphenols on various models of diabetes mellitus have been displayed.^9^ Polyphenols are a great and inaccordant group of phytochemicals of plant foods, including tea, berries and grape. Studies demonstrated that vegetarian diet is accompanied with reduced risk of important chronic diseases such as diabetes and cardiovascular diseases.^10^ Grape seed proanthocyanidins (GSPs), extracted from grape seeds, are known as a group of proanthocyanidins mainly containing dimers, trimers and other oligomers of catechin and epicatechin and their gallic acid esters. Studies of in vitro indicated that antioxidant effects of GSPs were more powerful than vitamin C and vitamin E. Also, anti-hyperglycemic property of GSPs in experimental studies on type 1 diabetes mellitus was shown.^11^ Recently, in vitro studies have shown that procyanidins protect against free radical mediated cardiovascular and renal disorders.^12^ Therefore, in the present study the effects of grape seed proanthocyanidin was investigated on the diabetic rats heart.

## Materials and Methods

 All chemicals used in the experiment were purchased from Sigma-Aldrich Chemical Co. (St. Louis, USA), unless otherwise stated.

Animals and induction of diabetes mellitus. A total number of 40 male Sprague Dawley rats (weighing 150 to 170 g) were prepared from animal house central of Ahvaz Jundishapur University of Medical Sciences, Ahvaz, Iran were used in this study in accordance with the Ethics Committee of Ahvaz University of Medical Sciences. Animals were maintained under controlled conditions of temperature (21 ± 2 ˚C) and a 12/12 hr light/dark cycle. They were fed normal rat diet and water. The animals were fasted overnight and diabetes was induced by a single intra-peritoneal injection streptozotocin (STZ) 50 mg kg^-1^ body weight) dissolved in citrate buffer (0.1 M, pH 4.5). Three days after STZ injection, fasting blood glucose levels were determined with a glucose strip test in a glucometer (Infopia Co., Anyang, Korea). Rats with blood glucose levels above 250 mg dL^-1^ were defined as the diabetic animals.

Experimental design. The rats were divided into four groups of 10 animals each including Group I: control group was allowed normal rat diet and water, Group II: control group were given GSP (95% purity, Hangzhou Joymore Technology Co., Zhejiang, China) dissolved in normal saline orally (200 mg kg^-1^), Group III: diabetic group, and Group IV: diabetic group treated with GSP (200 mg kg^-1^). The treatment was started on the fourth day after the STZ injection and this was considered as the first day of treatment. The treatment was continued daily for four weeks.

Blood pressure determination. Systolic blood pressure of conscious rats were measured by the tail cuff method using Power lab 4.0 channels (AD Instrument, Sydney, Australia). Briefly, rats were placed in clear acrylic cages and were consistent with the cage for 15 min, then was used of the tail pressure cuff with a diameter of about 1.5 cm and length of 3 cm. The rat tail was placed in the cuff and rat tail pressure was measured three times. The mean of different values of ​​arterial pressure was considered as systolic pressure. The first pulse after the opening of tail cuff indicated the systolic pressure.

Sampling and sample processing procedures. At the end of 4^th^ week, six rats were selected randomly from each group then overnight-fasted rats were scarified under ether anesthesia and blood samples were obtained by cardiac puncture and collected into heparinized tubes for determination of glycosylated hemoglobin (HbA1c) and preparation of plasma. Then, the hearts were removed and rinsed with isotonic saline and then blotted dry and weighed after weighing, the heart tissue was minced and a homogenate was prepared with 5% (w/v) potassium phosphate buffer (0.1 M, pH 7.4) using a homogenizer (Model silent crusher-M; Heidolph Instruments, Donau, Germany). Homogenate was then centrifuged at 16,000 g for 20 min to remove nuclei and cell debris. These supernatants were used for the measurement of lipid peroxidation and antioxidant enzymes activities.

Glycosylated hemoglobin and glucose levels measurement. Blood glucose levels were measured by applying glucoseoxidase method using diagnostic kit (Pars Azmoon Co., Tehran, Iran) by spectrophotometer. The HbAlc was estimated by a commercial Kit (BioSystem SA, Barcelona, Spain) according to manufacturer method.

Lipid peroxidation determination. Lipid peroxidation was estimated by malondialdehyde measurement in terms of thiobarbituric acid reactive substances (TBARS) formation.^13^ A volume of 500 µL of supernatant was added to 1.5 mL of 10% trichloroacetic acid (TCA) and centrifuged at 4,000 g for 10 min, then 1.5 mL of supernatant was mixed with 2 mL thiobarbituric acid (TBA) (0.67%) and heated for 30 min at 100 ˚C. After cooling, 2 mL of n-butanol was added to samples and centrifuged at 4,000 g for 15 min, Finally, absorbance of the resulting pink chromophore was determined at 532 nm using spectrophotometer (Model UNICO UV-2100; United products and instruments Inc., Dayton, USA). Values were expressed as nmol per mgprotein. The TBARS concentrations of the samples were calculated from a standard curve using 1,1,3,3-tetramethoxypropane.

Determination of antioxidant enzymes activities. Analyses of heart antioxidant enzyme activities were accomplished by spectrophotometer. Catalase (CAT): activity was measured by the method described by Claiborne.^14^ The final reaction volume of 1 mL contained 50 mM potassium phosphate (pH 7.0), 19 mM H_2_O_2_, and a 20-50 µL sample. The reaction was initiated by addition of H_2_O_2_, and absorbance changes were measured at 240 nm for 30 sec. Catalase activity was estimated using molar extinction coefficient of 43.6 M^1^ cm^-1 ^for H_2_O_2_. The level of CAT was expressed in terms of µmol H_2_O_2_ consumed min^-1^ per mg protein. Superoxide dismutase (SOD) and glutathione peroxidase (GPX) activities were determined using the diagnostic kits RANSOD and RANSEL (Randox Laboratories, Crumlin, UK) were expressed in unit per mg protein.

Protein determination. Protein was assayed using method of Bradford.^15^ In brief, bovine serum albumin was used as standard. A volume of 20 μL of sample or standard was added to 1mL Bradford reagent (BioRad, Cambridge, UK), and then the absorbance was read by a spectrophotometer at 595 nm after 5 min.

Statistical analysis. Data were expressed as the mean ± SE. Statistical significance of differences was assessed with one-way ANOVA by SPSS (Version 18; SPSS Inc., Chicago, USA) followed by Tukey’s test. A p value less than 0.05 was considered as statistically significant.

## Results

Body and heart weight. As shown in the Table 1, body weight was decreased significantly in diabetic group when compared with the control (Group I). However, treatment by GSP (Group IV) significantly suppressed weight loss caused by diabetes in group II. In diabetic group, the heart weight was increased significantly compared to control group. Administration of GSP (Group IV) significantly reduced the heart weight of rats compared to diabetic group rats, (Table 1).

**Fig. 1:**
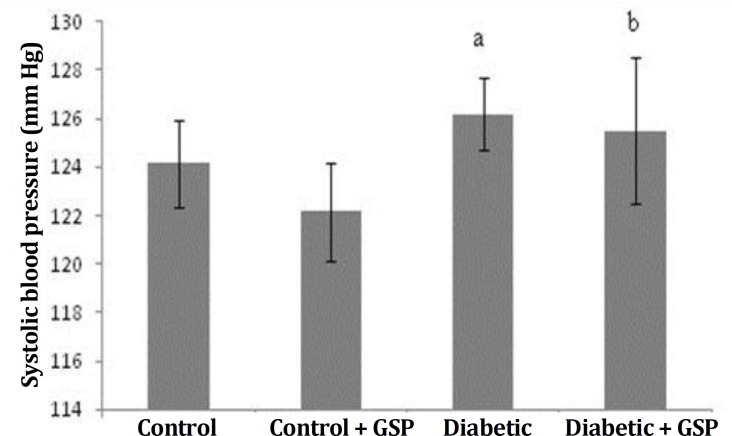
Effect of GSP (grape seed proanthocyanidin) on systolic blood pressure in different groups. I: Control, II: Control + GSP (200 mg kg^-1^), III: Diabetic, and IV: Diabetic + GSP (200 mg kg^-1^). Results are expressed as mean ± SE (n = 6).

^a^ indicates no significant difference compared to control(p > 0.05).^b^ indicates no significant difference compared to diabetic group (p > 0.01).

Systolic blood pressure (SBP). There was no considerable variations in SBP level between the groups (Fig. 1).

**Fig. 2:**
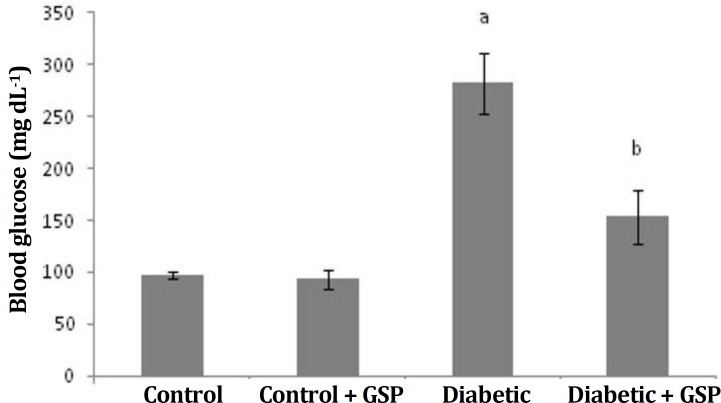
Effect of grape seed proanthocyanidin on blood glucose in different groups. I: Control, II: Control + GSP (200 mg kg^-1^), III: Diabetic, and IV: Diabetic + GSP (200 mg kg^-1^). Results are expressed as mean ± SE (n = 6).

^a,b^ indicate statistically significant difference compared to control group and diabetic group, respectively (p < 0.001).

**Fig. 3:**
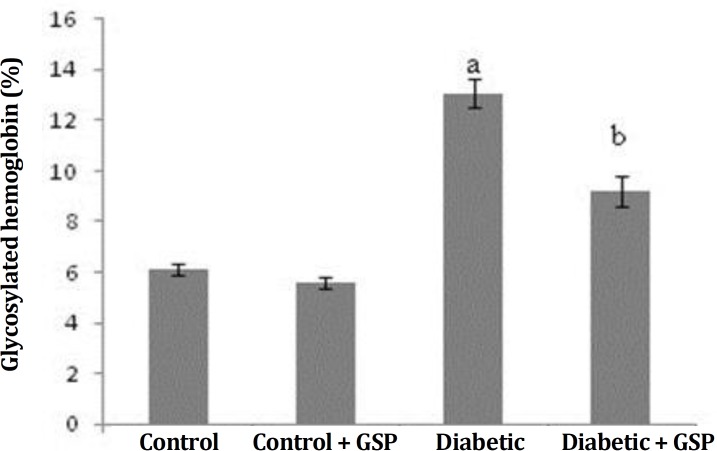
Effect of grape seed proanthocyanidin (GSP) on glycosylated hemoglobin in different groups. I: Control, II: Control + GSP (200 mg kg^-1^), III: Diabetic, and IV: Diabetic + GSP (200 mg kg^-1^). Results were expressed as mean ± SE (n = 6).

^a,b^ indicate statistically significant difference compared to control group and diabetic group, respectively (p < 0.001).

HbA1c levels. Blood glucose concentration and HbA1c level in diabetic rats were significantly higher than control rats (Group I). After treatment with GSP, diabetic rats (Group IV) displayed significantly decreased (p *<* *0.005*) plasma glucose and HbA1c levels, close to the normal levels (Figs. 2 and 3).

**Table 1:** Effects of grape seed proanthocyanidin (GSP) on weight of body and heart in experimental groups (n = 6). Values are expressed as mean ± SE.

**Groups**	**Body weight (g)**	**Heart weight (g)**
**I (** **Control)**	271.16 ± 3.37	0.78 ± 0.03
**II (Control + GSP)**	273.33 ± 5.45	0.81 ± 0.06
**III (Diabetic)**	190.33 ± 6.36^a^	1.05 ± 0.06^a^
**IV (Diabetic + GSP)**	230.83 ± 4.62^b^	0.82 ± 0.05^b^

a,b indicate statistically significant difference compared to control group and diabetic group, respectively (*p* < 0.05).

**Fig. 4:**
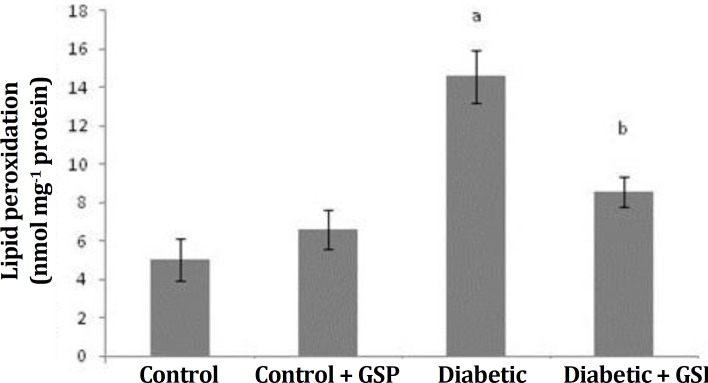
Effect of grape seed proanthocyanidin (GSP) on Lipid peroxidation in different groups. I: Control, II: Control + GSP (200 mg kg^-1^), III: Diabetic, and IV: Diabetic + GSP (200 mg kg^-1^). Results were expressed as mean ± SE (n = 6).

^a,b^ indicate statistically significant difference compared to control group and diabetic group, respectively (p < 0.001).

Lipid peroxidation level. In diabetic rats, lipid peroxidation was significantly increased (p < 0.05) in the heart tissue compared to the control rats. Treatment of diabetic rats with GSP significantly decreased (p < 0.005) the concentration of lipid peroxidation heart tissue when compared to untreated diabetic rats, (Fig. 4).

**Fig. 5:**
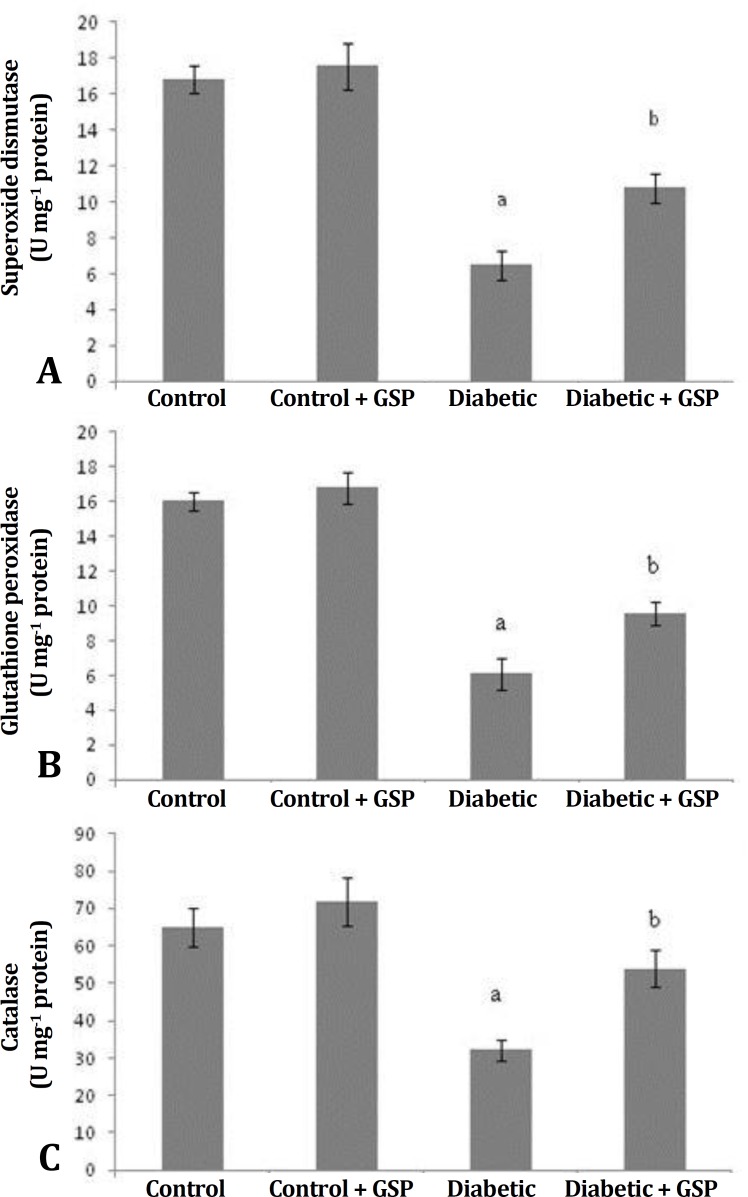
Effect of grape seed proanthocyanidin (GSP) on antioxidant enzymes (A: Superoxide dismutase, B: glutathione peroxidase and C: catalase) in different groups. I: Control, II: Control + GSP (200 mg kg^-1^), III: Diabetic, and IV: Diabetic + GSP (200 mg kg^-1^). Results were expressed as mean ± SE (n = 6).

Antioxidant enzymes activities. The effect of GSP on antioxidant enzymes activities like SOD, GPx and CAT were estimated (Figs. 5A, 5B and 5C). The activities of cardiac antioxidant enzymes were significantly decreased in diabetic rats. They indicated a significant increase in the diabetic rats treated with GSP in comparison with the control group. In all the above mentioned parameters there were no significant difference between diabetic rats receiving GSP (Group III) and control group rats (Group I).

 ^a,b^ indicate statistically significant difference compared to control group and diabetic group, respectively (p < 0.001).

## Discussion

Cardiovascular disease is one of the main complications of diabetes. Oxidative stress caused by diabetes has been involved in the development of pathogenesis of the cardiovascular complications and antioxidants were selected as hopeful therapeutic approach. In spite of the attendance of a lot of anti-diabetic pharmaceuticals factors, there is renewed attention to herbal medicine because of their less side effects and low costs.^3^^,^^16^ Diabetes caused by STZ is associated with intensive decline in body weight of diabetic rats, which is due to an increase muscle atrophy in diabetes,^17^ also this decrease may be due to deficiency or destruction of structural proteins because these proteins as the structural proteins contribute to body weight. In this study, a reduction in body weight was found in STZ-induced diabetic rats. Also, when GSP were administered to diabetic rats for four weeks, there were differences in body weight of the rats (Table 1). The change in body weight showed that GSP had a significant effect in controlling the loss of body weight, that happened during diabetes. Weight gain was probably due to the features of anti-hyperglycemia of GSP.^18^ In the diabetic rats, a significant increase in the heart weight was observed. It may be due to hyperglycemia induced myocardial hypertrophy and myocardial fibrosis,^19^ that probably is main factor for increase of heart weight. Results of the present study about body and heart weight were in agreement with previous studies.^20^^,^^21^ As was shown, there was no significant difference in SBP among the four groups. Like the previous study probably diabetes in the short term no effect on blood pressure.^22^ The GSPE had no effects SBP in diabetic group. This result was in agreement with the previous study.^23^ In the present investigation, diabetic animals showed increased levels of plasma glucose and HbA1c, compared to control rats. Diabetes mellitus is diagnosed by hyperglycemia due to handicap in insulin discharge or function or both.^24^ Thus, decreased levels of insulin leads to glycogenolysis, increase in glucose production and reduce the use of glucose by cells. Decreased insulin levels in STZ-induced diabetic rats were probably due to injury of beta cells and reduction of secreted insulin by these cells. STZ devastated the pancreatic cells selectively and induced hyperglycemia.^25^ Glycated hemoglobin rises in diabetic people with uncontrolled or poorly controlled blood sugar and is straightly proportional to the blood sugar level.^26^Studies suggested that glycation probably causes the formation of oxygen-induced free radicals in diabetic patients, thus the amount of HbA1c is used as one of the indicators of grade of oxidative stress in diabetes mellitus. Therefore, the estimate of HbA1c is considered as a very susceptible indicator for blood sugar control.^27^ This study indicated that treatment with GSP significantly reduced levels of HbA1c and plasma glucose in treated-diabetic rats. These findings were similar to previous study.^28^ The present study showed a significant increase of heart lipid peroxidation in diabetic rats. Decrease of circulating insulin in diabetes raises the activity of the enzyme fatty acyl Co-A oxidase that induces beta oxidation of fatty acids, leading to the production of lipid peroxidation. Incremented lipid peroxidation damage membrane task with reduction of membrane fluidity and change in function of enzymes and receptors binding to membrane. Lipid peroxidation crops are detrimental to cells and may causedifferentdiseases.^29^ In the current study, the SOD, CAT and GPx activities were significantly reduced in heart of diabetic rats. The reduced activities of antioxidant enzymes in heart during diabetes mellitus was probably due to the formation of reactive oxygen free-radical that can themselves decrease the activity of these enzymes. The SOD, CAT and GPx not only abolish the peroxides but also play a key role in preparing antioxidant protection to an organism. The SOD operates to dismutate superoxide radical to H_2_O_2_ then GPx and CAT are involved in the deletion of H_2_O_2_. The functions of all these enzymes are interrelated and their reduced activities lead to the accumulation of lipid peroxides and enhancement oxidative stress in diabetic rats.^26^ Treatment of GSP decreased the amount of lipid peroxidation and increased the activity levels of antioxidant enzymes, including SOD, CAT and GPx. Our observations are similar to the previous study.^30^ It can be concluded from the data that GSP is beneficial in controlling the blood glucose level, amelioration of the lipid metabolism and repair of antioxidant defense systems in the heart of diabetic rats. This could be useful for prevention or early treatment of cardiac disorders induced by diabetes.
